# Long COVID or Post-COVID-19 Condition: Past, Present and Future Research Directions

**DOI:** 10.3390/microorganisms11122959

**Published:** 2023-12-11

**Authors:** César Fernández-de-las-Peñas, Arkiath Veettil Raveendran, Rocco Giordano, Lars Arendt-Nielsen

**Affiliations:** 1 Department of Physical Therapy, Occupational Therapy, Physical Medicine and Rehabilitation, Universidad Rey Juan Carlos, 28922 Madrid, Spain; 2 Center for Neuroplasticity and Pain (CNAP), Center for Sensory-Motor Interaction (SMI), Department of Health Science and Technology, Faculty of Medicine, Aalborg University, DK-9220 Aalborg, Denmark; rg@hst.aau.dk (R.G.); lan@hst.aau.dk (L.A.-N.); 3 Govt. Medical College, Kozhikode 676121, Kerala, India; raveendranav@yahoo.co.in; 4 Department of Medical Gastroenterology, Mech-Sense, Aalborg University Hospital, DK-9000 Aalborg, Denmark; 5 Steno Diabetes Center North Denmark, Clinical Institute, Aalborg University Hospital, DK-9000 Aalborg, Denmark

**Keywords:** COVID-19, post-COVID, long-COVID, mechanisms, vaccine, reinfections, genetics

## Abstract

The presence of symptoms after an acute SARS-CoV-2 infection (long-COVID) has become a worldwide healthcare emergency but remains underestimated and undertreated due to a lack of recognition of the condition and knowledge of the underlying mechanisms. In fact, the prevalence of post-COVID symptoms ranges from 50% during the first months after the infection up to 20% two-years after. This perspective review aimed to map the existing literature on post-COVID symptoms and to identify gaps in the literature to guide the global effort toward an improved understanding of long-COVID and suggest future research directions. There is a plethora of symptomatology that can be due to COVID-19; however, today, there is no clear classification and definition of this condition, termed long-COVID or post-COVID-19 condition. The heterogeneity in the symptomatology has led to the presence of groups/clusters of patients, which could exhibit different risk factors and different mechanisms. Viral persistence, long-lasting inflammation, immune dysregulation, autoimmune reactions, reactivation of latent infections, endothelial dysfunction and alteration in gut microbiota have been proposed as potential mechanisms explaining the complexity of long-COVID. In such an equation, viral biology (e.g., re-infections, SARS-CoV-2 variants), host biology (e.g., genetics, epigenetics) and external factors (e.g., vaccination) should be also considered. These various factors will be discussed in the current perspective review and future directions suggested.

## 1. Introduction

The Severe Acute Respiratory Syndrome Coronavirus 2 (SARS-CoV-2), the pathogen responsible for the worldwide spread of coronavirus disease 2019 (COVID-19), has provoked one of the most important health crises of the current century. Over the course of the last three years, since the onset of the outbreak, the emergence of new several SARS-CoV-2 variants has perpetuated its relentless transmission, resulting in a staggering 768 million confirmed cases and nearly 7 million reported deaths globally [1]. Substantial efforts have been focused on managing COVID-19 symptoms at the acute phase [2,3], the introduction of vaccines for preventing the severe form of the disease and for decreasing mortality rates [4] and for preventing the spreading of SARS-CoV-2 variants [5].

A growing concern from the beginning of the pandemic has been also the development of long-lasting symptomatology once the acute phase of the infection has surpassed. From the beginning of the pandemic, more than 100 post-COVID symptoms affecting multiple systems, e.g., cardiovascular, neurological, respiratory and musculoskeletal, have been described [6]. The presence of long-lasting post-COVID symptoms is associated with reduced health-related quality of life [7], a substantial increase in healthcare utilization and increased direct and indirect medical costs [8]. In fact, the health-related quality of life of patients with long-lasting post-COVID symptoms is severely impacted and can be compared with that of people with chronic pain after spinal surgery [9]. 

Several meta-analyses have reported that up to 50% of individuals who had survived a SARS-CoV-2 acute infection exhibit a plethora of long-lasting symptoms from several weeks or months [10,11,12] up to one year after [13,14,15]. Thus, the Global Burden of Disease Long COVID study, which included 1.2 million subjects who had experienced an acute SARS-CoV-2 infection, reported that 51% of COVID-19 survivors suffered from at least one long-lasting post-COVID symptom the first three months after and that to up to 15.1% of subjects experience symptoms 12 months after [16]. A PubMed search conducted on 10 November 2023 revealed more than 2500 papers describing different post-COVID symptoms. Rahmati et al. observed that around 40% of COVID-19 survivors still experience at least one post-COVID symptom two years after the infection [17]. Fatigue (51%) [18], cognitive problems (25%) [19] and pain (20%) [20] are the most prevalent post-COVID symptoms. Overall, SARS-CoV-2 infection has been associated with a higher risk of developing post-COVID fatigue (RR 1.72, 95%CI 1.41, 2.10), dyspnea (RR 2.60, 95%CI 1.96, 3.44), memory difficulties (RR2.53, 95% CI 1.30, 4.93) and concentration difficulties (RR2.14, 95%CI 1.25, 3.67) [21].

It is important to consider the complex pathways that link SARS-CoV-2 acute infection, the putative biological mechanisms of the host and the heterogeneous phenotypes of post-COVID symptomatology [22]. The current perspective review aimed to map the existing literature on post-COVID symptomatology (from the definition of the condition to the underlying mechanisms (including genetics) and to identify gaps in the current literature to guide the global effort toward an improved understanding of long-COVID [23]. Each section reviews current literature and includes questions for future research.

## 2. How Should Post-COVID Symptoms Be Defined

Different terms have been used for defining the presence of symptoms persisting after the acute phase of a SARS-CoV-2 infection being post-COVID, long-COVID, post-COVID-19 condition, or post-acute sequelae of SARS-CoV-2 infection (PASC) are the most commonly used [24,25].

The World Health Organization (WHO) adopted the term post-COVID-19 condition: “post-COVID-19 condition occurs in people with a history of probable or confirmed SARS-CoV-2 infection, usually three months from the onset of infection, with symptoms that last for at least two months and cannot be explained by an alternative diagnosis. Common symptoms include, but are not limited to, fatigue, shortness of breath (dyspnea), and cognitive dysfunction, and generally have an impact on everyday functioning. Symptoms might be new onset following initial recovery from an acute COVID-19 episode or persist from the initial illness. Symptoms might also fluctuate or relapse over time” [26]. Several aspects of this definition should be considered. 

First, the definition is focused on three symptoms, and although it includes “not limited to”, it does not consider bothersome and prevalent symptoms such as pain. We believe that the presence of any symptom showing a temporal relationship with the SARS-CoV-2 infection (another aspect for clarification) should be included in the definition, independently of its prevalence. Although fatigue is by far the major post-COVID symptom, combined or as a singleton symptom [27], other post-COVID symptoms, e.g., dyspnea, have shown prevalence rates up to 26% [28]. Thus, one particular post-COVID symptom could have different repercussions in daily life activities depending on each particular patient, accordingly, personalized application of this definition must be provided. 

Second, this definition did not consider the presence of previous symptoms or medical co-morbidities before the acute infection. This is an important topic because COVID-19 can promote the development of new symptoms (new-onset post-COVID symptom, a symptom not experienced before the infection and appearing after) or can also worsen previous symptoms (exacerbated post-COVID symptom) [29]. The definition of post-COVID-19 condition includes “symptoms might be new-onset after initial recovery from an acute COVID-19 episode or persist from the initial illness (persistent post-COVID symptom, a symptom experienced by a patient at the acute phase and persisting after without remission periods)”. This aspect is related to the relapsing/remitting nature of post-COVID symptoms and the time existing between the appearance of the symptom in relation to the infection [30]. These two post-COVID symptoms are included in the main definition; however, a third type, delayed-onset post-COVID symptom (a symptom not experienced at the acute phase of the infection and appearing after a latency period in relation to the infection) has been also described, although questioned [30]. An explanation for a delayed-onset post-COVID symptom is that SARS-COV-2 can trigger latent neurodegenerative processes with residual damage on persistent immune activation or unmasking of underlying co-morbidities [31]. 

Third, the temporal relationship with the SARS-CoV-2 infection is one of the hottest topics for the diagnosis of post-COVID-19 condition, and most definitions have focused on this aspect. The National Institute for Health and Care Excellence (NICE) Guideline, the Scottish Intercollegiate Guidelines Network, and the Royal College of General Practitioners proposed a time four weeks after the infection [32], Baig proposed three weeks after [33], and Halpin et al. proposed 12 weeks after [34] for the diagnosis of post-COVID-19 condition. The WHO proposed a 12-week timeframe [26]. Determining the timeframe for the diagnosis of post-COVID-19 condition is critical for clinically identifying if the symptoms can or cannot be attributed to SARS-CoV-2 infection, but it is highly difficult from a clinical point of view due to the latency nature of some post-COVID symptoms and the neurotropism of SARS-CoV-2. 

Finally, the term long-COVID (which was the term proposed by the patients themselves in Spring 2020 as well as the term long-haulers [35]) was proposed for the presence, overall, of any post-COVID symptom after surpassing the SARS-CoV-2 infection [36]. In this definition, all potential situations associated with pre-existing symptomatology or medical conditions before the infection and different time frames are considered [36]. 

**Figure 1:**
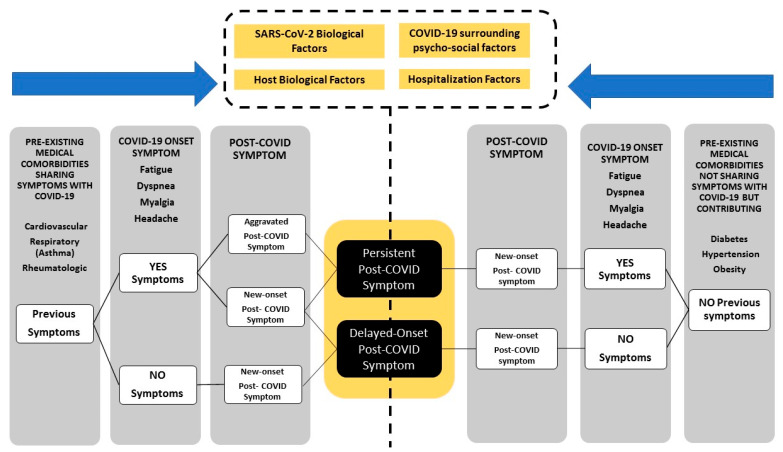
Proposal model for long-COVID symptomatology.

Figure 1 graphs the proposed development of post-COVID symptoms considering all these discussion points. First, if the patient reports the presence of a previous symptom, clinicians should differentiate if a particular post-COVID symptom is “new” or “exacerbated”. Similarly, the presence of a pre-existing medical co-morbidity who shares symptoms with COVID-19 should be also considered. In such a scenario, the temporality between the appearance of the symptom in relation to the acute infection should be investigated. If a symptom appears several months after the infection and is reasonably related (caused) by SARS-CoV-2, it should be considered as a post-COVID “delayed-onset” symptom. Second, the presence of a long-lasting post-COVID symptom that was also experienced at the acute phase of SARS-CoV-2 infection would lead to a “persistent” post-COVID symptom since this symptom started at the beginning of acute phase of infection and continue at post-infection without interruption. 

Future research direction: We do not currently know if this proposal would lead to different treatment outcomes or to a better classification of post-COVID symptomatology.

## 3. Subclassification (Clustering) and Phenotyping of Long-COVID

More than 100 symptoms could be attributed to SARs-CoV-2 infection [6]. It is clear that SARS-CoV-2 can affect all human systems: cardiorespiratory, gastrointestinal, neurological, dermatological, musculoskeletal, endocrine, visual and reproductive. Interestingly, research shows that patients with long-COVID tend to report post-COVID symptoms mostly associated with a particular system, leading to the hypothesis of the presence of clusters or subgroups. In fact, post-COVID clustering suggests different etiologies and mechanisms for each cluster.

The Global Burden of Disease Long-COVID study identified three clusters according to the most prevalent post-COVID symptoms: fatigue with pain in 51% of patients, respiratory symptoms in 60.4% of patients and cognitive problems in 35.4% of patients [16]. As it can be observed, the same patient could exhibit the combination of two clusters. Peter et al. found that the fatigue (37.2% of patients) and neurocognitive disturbances (31.3% of patients) groups were those mostly contributing to decreased health recovery whereas the musculoskeletal pain (16% of patients) group was the most disabled for working activities [37]. 

A recent meta-analysis has identified the following three clusters: (1) the cardiorespiratory cluster including fatigue, dyspnea, chest pain, muscle pain, headache, palpitations; (2) the systemic inflammatory cluster including dizziness, gastrointestinal symptoms, muscle pain, hair loss, muscle weakness and sleep disorders; or (3) the neurological cluster including headache, anosmia, paresthesia, neuropathies, dizziness, vision and balance problems, memory problems and poor concentration [15]. The estimated prevalence rates for the clusters were 36% (95%CI 32% to 40%) for the cardiorespiratory cluster (7 studies), 72% (95%CI 45% to 92%) for the neurological cluster (3 studies) and 46% (95%CI 17–77%) for the systemic inflammatory cluster (4 studies) [15]. As can be observed, pain symptomatology is present in all clusters identified by Kuodi et al. [15]. In fact, pain symptomatology is commonly found to be associated with the presence of fatigue or gastrointestinal problems in several chronic pain conditions; however, some authors have found that pain and fatigue can be also separated into two different clusters in individuals with post-COVID symptoms [38]. 

Future research direction: It is hypothesized that the different clusters/groups of patients with long-COVID could be associated with different underlying mechanisms; however, this has not yet been investigated. Future studies should “validate” the identified clusters by associating their symptoms with etiopathogenic mechanisms and treatment responses. 

## 4. Risk Factors Associated with Long-COVID 

Due to the catastrophic consequences of the COVID-19 outbreak, several attempts have been made to identify potential risk factors associated with the development of long-COVID. Initial evidence suggested that female sex, older age, a higher number of previous comorbidities, longer hospital stance, higher viral load, COVID-19 severity and a greater number of onset symptoms at the acute phase of the infection seem to be potential risk factors associated with long-COVID [39,40,41]. 

Different meta-analyses investigating risk factors associated with long-COVID have reported conflicting results. Maglietta et al. identified that the female sex was a risk factor for long-COVID whereas more severe SARS-CoV-2 infection was associated just with post-COVID respiratory symptoms [42]. Thompson et al. found that older age, female sex, white ethnicity, poor pre-infection health, obesity and asthma were associated with long-COVID [43]. Notarte et al. identified that the female sex and some specific comorbidities such as obesity were associated with long-COVID [44]. These authors observed that single studies reported that older age seems to be associated with long-COVID, but this association was not significant when they pooled data into their meta-analysis [44]. The most recent meta-analysis, conducted by Tsampasian et al. confirmed that female sex and obesity (higher body mass index) were associated with a higher risk of developing long-COVID [45]. This meta-analysis observed that older age, ICU admission and hospitalization were also associated with long-COVID [45].

To date, the only factor clearly associated with a higher risk of developing long-COVID is female sex [42,43,44,45]. Several biological and social factors can explain these sex differences. Interestingly, biological sex differences in the expression of angiotensin-converting enzyme 2 (ACE2) and transmembrane protease serine 2 (TMPRSS2) receptors can explain the higher survivor rate but a higher prevalence of long-COVID in female sex [46]. Thus, differences in physical capacity could also be involved. For instance, it has been recently observed that females who had been infected by SARS-CoV-2 exhibit a more reduced alveolar diffusion capacity or exercise tolerance than males [47]. In fact, although males and females have the same probability of being infected by SARS-CoV-2; males had higher rates of the severe form of COVID-19 and mortality than females [48,49,50,51]. Similarly, sex-dependent self-reporting bias should also be taken into account, considering the sex-dependent discrepancy in medical care already in place before the COVID-19 outbreak [27,52]. Accordingly, sex differences should be considered by clinicians when designing therapeutic strategies for a patient with long-COVID.

We could classify risk factors into: (1) host biological factors (those pre-infection features e.g., age, sex, pre-existing medical co-morbidities, previous health status, genetic predisposition); (2) SARS-CoV-2 biological factors (those associated with the infection e.g., disease severity, symptoms at onset, viral load, inflammatory response); (3) hospitalization factors (e.g., hospital stay, ICU admission, treatment received); and (4) surrounding psycho-social factors (e.g., stigmatization, sleep disorders, stressful situations, social influence, familiar problems).

Future research direction: It is possible that with the rapid increase in the number of long-COVID studies, all the risk factors have not yet been identified and further research is needed. For instance, the presence of depressive symptoms before the infection has been found to be a major risk factor for specific post-COVID symptomatology such as long-term dysautonomia as well as psychiatric symptoms in a single study [52].

## 5. Current Pathophysiology Theories of Long-COVID 

**Figure 2:**
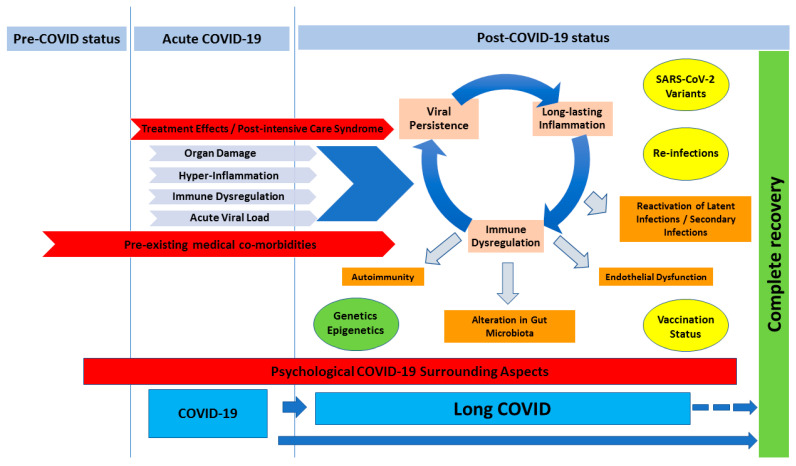
Potential pathophysiological mechanisms of long-COVID.

Long-COVID denotes symptoms and signs affecting multiple tissues and organ systems in the body. Thus, symptoms vary depending on the organ systems involved and the predominant pathophysiological mechanisms operating, making the diagnosis a challenge for clinicians [53]. In fact, pathophysiological mechanisms of long-COVID are not fully understood and different mechanisms are proposed and summarized in Figure 2 [54,55]. 

### 5.1. Viral Persistence 

Viral persistence is postulated as one of the most robust hypotheses for long-COVID [56]. It is possible that the SARS-CoV-2 pathogen may establish a persistent infection or leave non-infectious remnants in deep human tissues. Such a persistent reservoir or remnants will be able to generate pathogen-associated molecular patterns (PAMPs), such as viral RNA or bacterial cell wall and could engage host pattern recognition receptors (PRRs) triggering innate immune activation [57]. 

Several studies have investigated the presence of viral persistence or laboratory signs of persistent infection in people with long-COVID; nevertheless, heterogeneous results have been found [58]. It seems that persisting SARS-CoV-2 RNA is present in different human tissues but this persistence is time-dependent, i.e., there is a tendency to disappear with time from the infection, and also symptom-dependent, i.e., the virus is still present in the tissue associated with a specific post-COVID symptom. In fact, evidence supports that genes and proteins of the SARS-CoV-2 virus persist in the human body for variable periods (2–9 months) after the acute infection; however, data on viral persistence in follow-ups longer than one year are scarce [58]. Thus, SARS-CoV-2 RNA has been found in different human tissues such as the lungs [59], brain [60] or gastrointestinal tract [61], which could explain the presence of post-COVID dyspnea, brain fog and gastrointestinal symptoms, respectively. In addition, the presence of persistent SARS-CoV-2 spikes in plasma could lead to more systemic post-COVID symptoms such as chronic fatigue [62]. However, not all individuals experiencing post-COVID symptoms exhibit persistent SARS-CoV-2 RNA. It is possible that viral persistence is present in a range of 60% to 70% of individuals with long-COVID, but not in all. In fact, most published studies to date have not included a control (comparison) group of individuals who had been infected but did not develop persistent symptoms after SARS-CoV-2 acute infection [58]. Most studies have identified the presence of viral persistence in different tissues in small samples of patients with long-COVID but it has not been demonstrated yet if the same results would be observed in individuals without symptoms. 

Future research direction: If viral persistence is observed in a significant proportion of patients with long-COVID, it would justify the application of antivirals for the management of post-COVID symptomatology. Evidence supports the use of antivirals such as Nirmatrelvir-Ritonavir during the COVID-19 acute phase [63]. However, no published study has investigated the effect of antivirals in the management of patients with long-COVID, although several trials are currently being conducted: https://clinicaltrials.gov/ct2/results?cond=Long+COVID&term=nirmatrelvir%2Fritonavir+&cntry=&state=&city=&dist= (accessed on 15 October 2023).

### 5.2. Long-Lasting Inflammation

Different types of inflammatory responses (e.g., specific SARS-CoV-2 responses, new-onset autoimmune responses or a loss of normal immunoregulation) play an important role in the pathophysiology of acute COVID-19 [64]. In fact, long-lasting inflammation (systemic and tissue-specific) has been also found to be an important factor contributing to the development of long-COVID [65]. However, data about the level of inflammatory biomarkers, e.g., interleukins (IL) are conflicting, probably because inflammatory biomarkers were evaluated at two different moments, at hospital admission during the acute infection or months after the infection. Yin et al. reported that the levels of IL-6 are increased in individuals with long-COVID several months after the infection [66]. Similarly, a meta-analysis including up to 110 biomarkers also observed up-regulated IL-6, C-reactive protein and tumor necrosis factor-alpha levels in individuals with long-COVID [67]. This meta-analysis included studies evaluating the levels of these biomarkers at both the acute phase and the post-acute phase of the infection [67]. On the contrary, Williams et al. reported significant reductions in levels of different interleukins (IL-6, IL-2, IL-17, IL-13 and IL-4) in people with long-COVID, although the moment of extraction was not specified [68]. 

Other potential mechanisms can contribute to and perpetuate a long-lasting inflammatory state. For instance, since lymphocytes usually participate in inflammation resolution following an acute infection, lymphopenia associated with COVID-19 may promote this inflammation [69]. In addition, chronic inflammation can lead to the uncoupling of endothelial nitric oxide synthetase and reactivation of oxygen species production, which results in the endothelial dysfunction observed in individuals with long-COVID [70].

Future research direction: Identification of inflammatory biomarkers at a specific moment of the infection could be crucial for preventing (in the acute phase) or treating (in the post-COVID phase) long-COVID symptoms. The association of long-COVID symptoms with higher inflammation either in the acute phase of the infection or in the post-COVID phase would support the use of anti-inflammatory medications. Thus, the use of corticoids in the COVID-19 phase is supported by the current literature [71,72]; however, data on post-COVID are still lacking. Badenes Bonet et al. found that administrating Dexamethasone during the acute COVID-19 phase led to a shorter duration of long-COVID symptomatology in individuals with moderate or severe COVID-19 one year after infection [73]. Surprisingly, no trial investigating the effect of dexamethasone or corticoids for the management of long-COVID is registered: https://clinicaltrials.gov/search?cond=Long%20COVID&intr=DEXAMETHASONE, https://clinicaltrials.gov/search?cond=Long%20COVID&intr=Corticosteroids (accessed on 15 October 2023).

### 5.3. Immune Dysregulation and Autoimmunity

During the acute COVID-19 phase, B cell function is altered resulting in the production of autoantibodies against interferon, neutrophils, connective tissue, cyclic citrullinated peptides and cell nucleus [74]. Further, acute SARS-CoV-2 infection leads to T cell dysfunction and antigen-presenting cells present antigens to autoreactive T cells by bystander activation, resulting in the infiltration of CD8+ T cells in various organs similar to that seen in autoimmune conditions [75]. Autoimmune response to various self-antigens through molecular mimicry will result in various organ damage [76].

The specific and autoreactive immune response induced by SARS-CoV-2 could persist in some individuals even after recovery from the acute infection and contribute to long-COVID [77]. In fact, elevated CD4+ T cell response and exhausted CD8+ T cell response with a dysregulation between humeral and cellular immunity is found in people with long-COVID [78].

Future research direction: Since immune response is intrinsically linked to the host, identification of predisposing individuals who exhibit a reduced or excessive response against SARS-CoV-2 could lead to early identification of potential long-lasting immune dysregulation or autoimmunity. No study has investigated this hypothesis.

### 5.4. Reactivation of Latent Infections

Reactivation of underlying pathogens in the body, e.g., herpes viruses such as Epstein–Barr virus (EBV) and human herpesvirus 6 (HHV-6) results in the development of various symptoms and has been observed in people infected by SARS-CoV-2 [57]. In fact, reactivation of EBV in patients with long-COVID is associated with fatigue and cognitive dysfunction [79]. A recent meta-analysis has found that the prevalence of different active herpes viruses ranges from 18% to 41% in COVID-19 survivors and that this prevalence is higher in individuals who have suffered from severe COVID-19 [80]. However, the prevalence of all evaluated active herpesvirus infections was not significantly different between infected and non-infected individuals [80]. Based on current data, the reactivation of latent infection in predisposing individuals can be another factor contributing to the development of some post-COVID symptoms. 

Future research direction: Future studies should investigate the risk factors associated with the reactivation of latent infections in predisposing patients who have been infected with COVID-19. Thus, the time of reactivation, i.e., at the acute COVID-19 phase or at a post-acute phase, could be also relevant to identify the underlying mechanisms of this process. 

### 5.5. Endothelial Dysfunction 

Endotheliitis and thrombo-inflammation can occur at the acute COVID-19 phase and could persist even after recovery [81]. This is related to the fact that SARS-CoV-2 is able to penetrate the endothelial barrier, causing potential endothelial cell injury, hyperinflammation cytokine storm syndrome, glycocalyx disruption, hypercoagulability and thrombosis [82]. In fact, oxidative stress, another process commonly observed during COVID-19 illness, also promotes endothelial damage, which increases the risk of long-COVID symptoms [83]. Thus, endothelial dysfunction and vascular damage of the brain could explain long-term post-COVID cognitive symptoms [84]. Based on current data, endothelial dysfunction can explain the heterogeneous cardiovascular post-COVID symptomatology experienced by patients, a condition called “vascular long-COVID” [85].

Future research direction: Future studies investigating if endothelial dysfunction in specific organs could be associated with a higher risk of developing particular post-COVID symptoms. Thus, early identification (in the acute COVID-19 phase if possible) of patients developing endothelial dysfunction could lead to better therapeutic strategies for preventing long-lasting symptoms. 

### 5.6. Alteration in Gut Microbiota

Microbiota dysbiosis contributes to the onset and progression of several viral diseases, including COVID-19, which could serve as potential diagnostic and prognostic biomarkers [86]. Gut dysbiosis or alteration in the gut microbiome during the acute COVID-19 phase results in increased inflammation, prolonged fecal shedding of SARS-COV-2 and dysfunction of the microbiota-gut-brain axis, which can degrade the intestinal barrier and increase the permeability to harmful substances [87]. This gut dysbiosis can explain, not only gastrointestinal symptoms seen at the post-COVID phase but also post-COVID cognitive symptoms [87]. In fact, Liu et al. observed that gut pathogens such as Clostridium innocuum or Actinomyces naeslundii were also correlated with respiratory long-lasting post-COVID symptoms [88].

Future research direction: As with previous underlying mechanisms associated with long-COVID, early identification (at the acute COVID-19 phase if possible) of patients developing gut dysbiosis could lead to better therapeutic strategies for preventing long-lasting symptoms. For instance, it seems that COVID-19 can trigger irritable bowel syndrome [89]. In such a scenario, it is also possible that predisposing subjects with previous irritable bowel syndrome who are infected by SARS-CoV-2 can develop more long-lasting gut dysbiosis and, thus, more long-COVID symptomatology. 

### 5.7. Psychological COVID-19 Surrounding Aspects 

Stress events associated with the COVID-19 pandemic such as quarantine, social isolation, loss of friends or family, psychological disorders such as anxiety and depression, work stress, financial stress and post-traumatic stress disorders can promote or exacerbate long-COVID symptoms. 

The presence of emotional aspects in people with long-COVID supports that biological and psychosocial factors interact in this complex condition [90]. The presence of mood disorders such as anxiety and depressive symptoms as well as sleep disturbances in individuals with long-COVID is supported by the current literature [41,91]. It has been found that depressive symptoms are associated with a higher risk of post-COVID fatigue [92] or dyspnea [93]. It has been hypothesized that sleep problems can perpetuate long-COVID symptomatology [94]. Thus, it is clinically seen that the interaction between anxiety, depression and sleep problems is complex in people with long-COVID [95]. Accordingly, the presence of associated psychological factors could promote or perpetuate long-COVID symptomatology.

Future research direction: It is possible that the personality traits of the patient could be risk factors for developing long-COVID symptomatology. We do not currently know the role of previous psychological disturbances due to the rapid spread of the main outbreak. 

## 6. Genetic Influence on Long-COVID 

With the introduction of high-throughput innovations such as genome-wide DNA-sequencing, imaging and bid data, it is accepted that the heterogeneity of many diseases requires treatment strategies targeting specific mechanisms—what has been called precision medicine [96]. Precision medicine refers to the ability to classify patients into subgroups that differ in their susceptibility to, biology, or prognosis of a particular disease or in their response to a specific treatment [97]. In such a scenario, genetic and epigenetic advances play a relevant role in precision treatment. For instance, since SARS-CoV-2 binds to human cells via ACE2 receptors, this receptor should be considered a vital medication target for managing COVID-19 [98]. At the beginning of the pandemic, early administration of non-steroidal anti-inflammatory drugs (NSAIDs) at the acute COVID-19 phase was contraindicated, since it was hypothesized that NSAIDs could interact with ACE2 receptors. Current research reveals that several NSAIDs do not exhibit any effects on ACE2 expression or activity [99]. 

Several studies have investigated long-COVID putting forth evidence that genetic elements may contribute significantly to this phenomenon, opening further comprehensive research into underlying mechanisms. Previous research has extensively looked into the potential role of genetics and epigenetics in determining an individual’s predisposition to viral infections, as well as the severity and aggressiveness of such infections [100]. Genetic variations, including single nucleotide polymorphisms (SNPs), have demonstrated their involvement at various levels in viral infection processes [101]. These variations in SNPs influence diverse genes implicated in the regulation of viral pathogenesis and the initiation of immune response pathways within the infected host [101]. In the context of SARS-CoV-2 infection, multiple studies have evaluated the effects of variations in several genes concerning viral activity, increased infection risk and protective effects against its actions. The most investigated genes related to COVID-19 include ACE1, ACE2 and TPRSSM2 due to the functions of their products, which have been proven to be involved in the cell invasion process and cleavage and activation of the SARS-CoV-2 virus spike protein [102]. The genetic modification of ACE1, ACE2, or TMPRSS2 can elicit beneficial or protective outcomes against SARS-CoV-2 [103,104].

Extensive research has been also conducted on genetic variations of genes implicated in inflammatory and immune responses. Particular attention has been given to evaluating gene variations of interferons, a specialized family of cytokines crucial for initiating antiviral responses and activating the host’s defense mechanisms against external viral infections [105]. These investigations revealed that certain gene variants of interferon-induced transmembrane protein SNPs were identified in subjects suffering from severe SARS-CoV-2 pneumonia, showing an association with COVID-19 severity [106]. Thus, genotyping of cytokine-related polymorphisms, such as IL-6, in the context of COVID-19 has also been conducted, although the association between IL-6 polymorphisms and SARS-CoV-2 pathogenesis and severity has not been firmly established [107]. It has been suggested that IL-6 polymorphisms should be considered as a critical factor in the development of targeted therapies against COVID-19 [107].

Our research group aimed to identify an association between some candidate genes and the development of long-COVID symptoms. The results did not reveal an association between SNPs of ACE2 and TMPRSS2 genes (involved in SARS-CoV-2 pathogenesis) and the development of long-COVID symptomatology [108]. Similarly, no association has been identified between SNPs of genes regulating the inflammatory (e.g., IL-6) [109] or the pain (e.g., COMT) [110] response and post-COVID pain. It is important to highlight that the reported results are based on an individual cohort. 

Future research direction: A more comprehensive evaluation that encompasses a larger and diverse population of individuals with long-COVID symptoms could potentially mitigate the inherent genetic variability and lead to the identification of genetic variations associated with this symptomatology. Additionally, the inclusion of a large number of SNPs and genes can also elucidate potential relationships with specific post-COVID symptoms.

## 7. Epigenetic Influence on Long-COVID 

Together with genetic alterations, epigenetics plays a major role in the natural gene expression and pathophysiological phenotype and has also been investigated in relation to viral infections [111]. 

Epigenetics is defined as the molecular process that regulates gene expression without altering the DNA sequence including methylation, histone protein modifications and the action of non-coding RNA (ncRNA) [112]. These molecular changes can be influenced by several internal and external factors, such as environmental exposures, stress and nutrition, leading to a specific regulation of protein product production [113]. The effects of epigenetic changes induced by COVID-19 infection are still under investigation, with research looking into possible systemic and cellular modifications induced by SARS-CoV-2 infection [114]. Several research studies have been conducted to investigate methylation patterns in COVID-19 patients [115]. Most research reveals a distinct pattern of hypermethylation in interferon-related genes and hypomethylation in genes responsible for regulating the inflammatory response providing valuable evidence regarding the dynamic epigenetic regulation of genes that play crucial roles in determining COVID-19 severity [116]. Additionally, recent findings have demonstrated that the methylation signatures detected during the acute phase of infection persist even after one year from the initial infection, specifically in circulating leukocytes [117].

Further, an Epigenome-Wide Association Study (EWAS) reported a hypomethylation of three specific CpG islands located on the gene Interferon-induced protein 44 like (IFI44L), involved in the interferon-induced innate viral response and protection against disease, in COVID-19 patients three months after the infection, showing not a clear association with the severity of the infection but reporting the possibility to detect this alteration after some time and hypothesizing an involvement of interferon responsive genes in the pathophysiology of COVID-19 and indicate a possible link to systemic autoimmune diseases [118]. Other epigenetic changes, such as epigenetic aging driven by telomers regulators of cell senescence, have been shown to increase susceptibility to SARS-CoV-2 infection and severe COVID-19 [119]. This molecular mechanism is possibly influenced by SARS-CoV-2 itself, leading to accelerated epigenetic aging and contributing to long-COVID [119]. Epigenetic changes have been also proposed as a potential therapy for COVID-19, with the action of miRNA, derived from mesenchymal stem cells (MSCs) able to block the action of IL-6 demonstrated to be a biomarker of long-lasting post-COVID symptoms, such as fatigue, depression and anxiety in COVID-19 survivors [120,121].

Regarding the identification of biomarkers for the individualization of long-COVID patients, recent systematic reviews have brought attention to a potential subset of serological biomarkers that warrant investigation in the circulation of people with long-COVID. These reviews found elevated levels of inflammatory biomarkers and cytokines, such as C reactive protein (CRP), IL-6, D-dimer, lactate dehydrogenase (LDH) and TNF-α in people with long-COVID [67,122]. Furthermore, in the context of epigenetic changes, miRNA regulation during different stages of acute SARS-CoV-2 infection demonstrated its potential to help in patient stratification allowing for the estimation of heightened risk of long-term health issues following the acute phase [123]. These miRNA profiles also hold promise for identifying informative biomarkers that can be targeted for preventive measures.

Future research direction: Despite the increasing evidence, there is still a lack of results that validate the epigenetic involvement in individuals with long-COVID. In this regard, future studies that explore and compare these processes in various affected populations will provide insight into the effects of this recent pandemic and highlight promising potential targets for understanding long-COVID.

## 8. SARS-CoV-2 Variants, Re-Infections and Vaccination Status

The fast spread of SARS-CoV-2 resulted in the appearance of several variants in a short period of time [124]. Among all SARS-CoV-2 variants identified after the historical strain (20A.EU2), Alpha (B.1.1.7), Delta (B.1.617.2) and Omicron (B.1.1.529/BA.1) are considered those variants of concern (VOCs) [125]. Current data suggest that the prevalence of long-COVID symptomatology is higher in individuals infected with the historical strain when compared with posterior SARS-CoV-2 variants (e.g., Alpha, Delta, Omicron) and that the risk of long-COVID seems to be lower in individuals infected with the Omicron variant [126,127]. Nevertheless, the presence of these variants has led to an increased number of reinfections. Thus, SARS-CoV-2 reinfections were uncommon until the end of 2021 (when the Delta variant was the predominant) but exponentially increased with Omicron variant (the most transmissible variant) [128]. 

Although individuals who had been infected with the historical strain had protection against reinfections with other SARS-COV-2 variants such as Alpha or Delta, this protection is lower with the Omicron variant [129]. Secondary infections are common due to organ damage and altered immune status. In fact, evidence shows that SARS-CoV-2 reinfection further increases the risks of death, hospitalization and organ damage [130]. Accordingly, it could be expected that re-infection with different SARS-CoV-2 variants could lead to a higher risk of developing long-COVID in those who did not develop long-lasting symptoms after the first infection, could exacerbate current symptoms or result in the appearance of new symptoms in individuals who developed long-COVID after the first infection [131]. In fact, Peghin et al. have observed that two years after the COVID-19 outbreak, the long-COVID dynamic seems to be not influenced by SARS-CoV-2 immunization status and reinfection [132], although there is evidence that pre-existing humoral immunity protects against long-COVID-19 [27].

In parallel with the appearance of SARS-CoV-2 variants, the development of vaccines has also marked the progression of the disease. It seems clear that vaccinated subjects had a significantly lower likelihood of reinfection than unvaccinated individuals [133]. Accordingly, the topic of reinfections could be prevented by vaccination. However, the effect of SARS-CoV-2 vaccines in long-COVID is different depending on whether vaccination has occurred before or after infection. Notarte et al. found a low level of evidence suggesting that administrating the vaccine before SARS-CoV-2 infection would reduce the risk of long-COVID [134]. However, the effect of vaccination in people with pre-existing long-COVID was not clear [134]. The meta-analysis by Gao et al. confirmed that vaccination had a protective effect against long-COVID if two doses are administered [135]. These authors also found that vaccination was effective against long-COVID independently if the vaccine is administered before or after SARS-CoV-2 acute infection [135]. A more recent meta-analysis confirmed that vaccination before SARS-CoV-2 infection is associated with a lower risk of long-COVID, but that vaccination in patients with ongoing long-COVID is not clear since most studies did not report any change in symptoms [136]. The fact that the impact of vaccines is higher when administered before infection should be expected since most people received the first two doses of COVID-19 vaccines in the first semester of 2021 when the Alpha and Delta SARS-CoV-2 variants were circulating. It therefore makes sense that vaccinated individuals were protected against severe COVID-19 caused by the Alpha and Delta variants and accordingly, lower rates of long-COVID would be expected. However, hybrid immunity did not provide further protection compared to vaccination or natural infection [27].

Future research direction: There is a lack of studies specifically investigating the development or changes in long-COVID symptomatology depending on the number of reinfections and on the specific SARS-CoV-2 variant [137]. Studies considering reinfections and vaccination status are needed to clarify this. 

## 9. Conclusions

Although there has been an increasing advance in the understanding of long-COVID in the recent two years, it remains a not well-understood, underestimated and undertreated condition due to the lack of recognition of the phenomenon and proper knowledge of underlying mechanisms. The current paper discusses past, present and future research directions of long-COVID in different aspects: (1) definition and phenotyping; (2) identification of sub-groups (clusters); (3) risk factors; (4) underlying pathophysiological mechanisms; (5) genetics and epigenetics, and (6) reinfections by SARS-CoV-2 variants and vaccination status. Each section of the current perspective review includes some questions to be answered in future studies based on the identified gaps in the current literature.

## Data Availability

Not Applicable.
